# Enhanced meta-heuristic optimization of resource efficiency in multi-relay underground wireless sensor networks

**DOI:** 10.7717/peerj-cs.1357

**Published:** 2023-04-27

**Authors:** Mariem Ayedi

**Affiliations:** Department of Computer Science, College of Computer Engineering and Sciences, Prince Sattam Bin Abdulaziz University, Al-kharj, Saudi Arabia

**Keywords:** Multi-relay underground wireless sensor networks, Resource efficiency, Relay selection, Chaotic theory, Crossover algorithm, Salp swarm algorithm

## Abstract

Achieving a balanced energy and spectral resource utilization is an interesting key design to extend the lifetime of underground wireless sensor networks (UWSNs) where sensor nodes are equipped with small limited energy batteries and communicate through a challenging soil environment. In this article, we apply an improved meta-heuristic algorithm, based on the Salp Swarm Algorithm (SSA), for multi-relay UWSNs where cooperative relay nodes amplify and forward sensed data, received from the buried source nodes, to the aboveground base station. Hence, the optimal nodes transmission powers, maximizing the network resource efficiency, are obtained and used to select beneficial relay nodes. The algorithm enhances the standard SSA by considering the chaotic map for salps population initialization and the uniform crossover technique for salps positions updates. Simulation results show that the proposed algorithm significantly outperforms the SSA in resource efficiency optimization and network lifetime extension. The obtained gain increases when the number of cooperative relay nodes increases. Furthermore, simulations prove the efficiency of the proposed algorithm against other meta-heuristic algorithms.

## Introduction

Underground wireless sensor networks (UWSNs) are an interesting solution for many applications in the underground world including mine environments observation, soil conditions monitoring, earthquake prediction and object localization (Akyildiz & Stuntebeck, 2006). In these networks, buried sensors collect, in real time, sensitive data regarding the underground conditions and forward it to the base station (Zhou, 2019). The primary factor, limiting the exploration and the evolution of UWSNs, is the complexity of the underground transmission link, which is mostly influenced by the heterogeneous soil medium consisting of sand, rocks, and watersheds. Moreover, the limited communication range and the difficulty of nodes batteries recharging are additional challenges of UWSNs (Akyildiz & Stuntebeck, 2006). Since sensor nodes cannot always communicate directly with aboveground base station, the role of relay nodes is important for achieving high bandwidth and expanding the network connectivity (Zhang et al., 2019). Relay nodes exploitation in UWSNs has been widely studied (Tam et al., 2020; Wu et al., 2014a; Yuan, Chen & Yao, 2017; Sharma & Prakash, 2020). Tam et al. (2020) divided the underground coal mine into separate regions and addressed the optimal relay node placement in order to enhance the coverage of the network. Wu et al. (2014a) aimed to control the amount of energy consumed by the underground sensors to map water pipelines *via* optimization of relay nodes placement. Also, the optimal relay node location was studied in Yuan, Chen & Yao (2017) to extend the network duration and to reduce the load balance and the number of relays. In Sharma & Prakash (2020), two approximation algorithms for assignment of relay nodes to sensor nodes are proposed with the aim to reduce the transmission loss among different nodes. Since obtaining energy efficiency (EE) gains is mostly achieved with sacrifices in spectrum efficiency (SE), research works in WSN concentrate recently on studying the trade-off between spectral efficiency and energy efficiency called the resource efficiency (Tang et al., 2014; Wei et al., 2015; Tang et al., 2015; Wu et al., 2014b). The main objective is to minimize the energy consumption jointly with efficient use of a limited frequency spectrum. In UWSNs, this problem is firstly addressed in Ayedi, Eldesouky & Nazeer (2021), where optimal source and relay powers used to transmit data to an aboveground base station, are computed in order to maximize the network resource efficiency. Ayedi, Eldesouky & Nazeer (2021) proposes a power allocation algorithm that exploits the Salp Swarm Algorithm (SSA) (Mirjalili et al., 2017) for solving the considered problem. The SSA is recently suggested in Mirjalili et al. (2017) as a novel meta-heuristic algorithm which outperforms many other meta-heuristic algorithms through tests on 19 different benchmark functions. In WSN research field, SSA demonstrates its efficiency in node localization optimization (Kanoosh, Houssein & Selim, 2019; Shi et al., 2019) and energy with lifetime optimization (Syed & Syed, 2019). The research (Ayedi, Eldesouky & Nazeer, 2021) proves that the SSA-based scheme offers a better resource efficiency compared to that of the fixed-powers conventional UWSN scheme. Ayedi, ElAshmawi & Eldesouky (2022) propose to further enhance the resource efficiency of the UWSN considered in Ayedi, Eldesouky & Nazeer (2021) by improving the SSA. A novel algorithm called Hybrid Chaotic Salp Swarm with Crossover (HCSSC) was proposed. The proposed algorithm uses the chaos theory (Tang, Fong & Dey, 2018) to generate the initial population since it improves the convergence speed and accuracy of optimization algorithms by enhancing the population diversity (Bingol & Alatas, 2020). Recently, chaotic numbers replace random numbers and give better results in many real-world problems (Bingol & Alatas, 2016; Bingol & Alatas, 2023). Furthermore, the uniform crossover operator (Syswerda, 1989; Hussain, Muhammad & Sajid, 2018; Umbarkar & Sheth, 2015) is merged in the exploration phase of the optimization algorithm to accelerate the algorithm convergence to the final optimal solution. In this article, we propose to adapt the proposed approach in Ayedi, ElAshmawi & Eldesouky (2022) to the multi-relay UWSN case where multiple cooperative relay nodes can help the source node to transmit data to the base station. Hence, the effect of the increase of the number of variables of the optimization problem is studied. For better accuracy, a three-dimensional node localization model is considered. Moreover, we propose that only beneficial relay nodes are selected to cooperate with the source node. The proposed power optimization scheme is tested for different relay nodes number and maximum allowed powers. Both fixed and variable relay nodes number among transmissions cases are studied. Further, the proposed algorithm is compared to other meta-heuristic algorithms, which are SSA, Artificial Bee Colony (ABC), Particle Swarm Optimization (PSO) and Dragonfly Algorithm (DA), in terms of resource efficiency, average remaining relay battery and performed number of transmissions.

The main contributions of this work are listed as follows:

(1) The HCSSC algorithm with relay selection scheme is proposed for multi-relay UWSN case.

(2) The optimal source and relay nodes powers are optimized and the resource efficiency performance is enhanced.

(3) The three-dimensional nodes localization case is studied.

(4) The proposed scheme is tested for different relay nodes number and different maximum allowed powers.

(5) The efficiency of the proposed algorithm is proved in case of both fixed and variable relay nodes number among transmissions.

(6) The superiority of the proposed algorithm against other meta-heuristic algorithms in maximizing the resource efficiency and extending the nodes batteries lifetime and the network longevity is demonstrated.

This article is structured as follows. In ‘UWSN System Model’, the UWSN system model is presented. In ‘Optimization problem’, the considered problem is formulated. In ‘Proposed Hybrid Chaotic Salp Swarm with Crossover Algorithm with Relay selection (HCSSC-RC)’, the proposed approach is detailed. In ‘Experimental Results and Analysis’, the experimental results and performance analysis are discussed. In ‘Conclusion’, conclusions are provided.

## UWSN System Model

The considered UWSN model consists of an underground source sensor node *S*, that gathers and forwards sensory data, to an aboveground base station *B* with the help of a set of *N*_*R*_ Amplify-and-Forward (AF) underground relay nodes which are within the communications range of *S*. Hence, each communication link is a two-hop link where the link between *S* and *R*_*v*_, *v* ∈ [1, *N*_*R*_] is an UnderGround-to-UnderGround (UG2UG) link, whereas the link between *R*_*v*_, *v* ∈ [1, *N*_*R*_] and *B* is an UnderGround-to-AboveGround (UG2AG) link. In this section, we, first, present the mathematical expressions of the path losses for UG2UG and UG2AG links. Then, we detail the communication mechanism among nodes.

### UG2UG-UG2AG channel model

**Figure 1:**
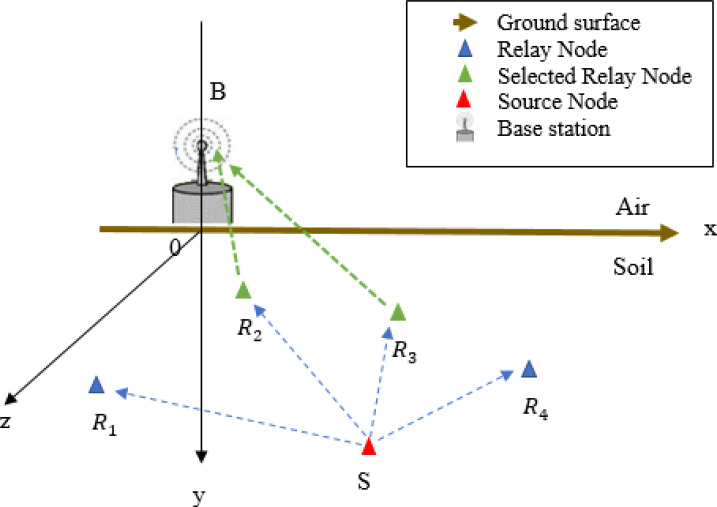
Topology of multi-relay UWSN.

In UWSNs, the source node is buried deeper than relay nodes according to the ground surface. As shown in Fig. 1, a three-dimensional (3D) space is considered. The location of each node *X* ∈ {*S*∪*N*_*R*_} is identified by its Cartesian coordinates *X*^*x*^, *X*^*y*^ and *X*^*z*^ measured from the origin *O*. The base station is positioned at the origin of *x*-axis, while, the ground surface, called *G*, represents the origin of *y*-axis and the horizontal distance between nodes is measured according to the *z*-axis. According to Vuran & Akyildiz (2010), the UG2UG path loss ℓ*uu*_*SR*_*v*__ of the link between *S* and *R*_*v*_, *v* ∈ [1, *N*_*R*_] is defined as follows

(1) \begin{eqnarray*}\ell {uu}_{S{R}_{v}}=6.4+20lo{g}_{10}({d}_{S{R}_{v}})+20lo{g}_{10}(\beta )+8.69\alpha {d}_{S{R}_{v}}-lo{g}_{10}(V)\end{eqnarray*}

where *d*_*SR*_*v*__ is the distance in metres between *S* and *R*_*v*_ given by

(2) \begin{eqnarray*}{d}_{S{R}_{v}}=\sqrt{({S}^{x}-{R}_{v}^{x})^{2}+({S}^{y}-{R}_{v}^{y})^{2}+({S}^{z}-{R}_{v}^{z})^{2}}.\end{eqnarray*}

The constants *α* and *β* measure the attenuation and the phase shifting, respectively. The factor *V* represents the attenuation of the reflection path obtained when the wave is reflected by the ground surface. Hence, the UG2UG communication results from propagation of the signal in the reflection path and in the direct path between sensors (Vuran & Akyildiz, 2010). Dobson et al. (1985) utilized an electromagnetic propagation model to provide the detailed expressions of the constants *α* and *β*. The soil medium dielectric characteristics along with the system operating frequency *q*, volumetric water content, percentages of sand and clay in soil, and bulk density constitute the main parameters on which these constants depend (Patitz, Brock & Powell, 1995). The soil and air constitute the two media throughout pass the communication between *R*_*v*_, *v* ∈ [1, *N*_*R*_] and *B*. However, there is no refraction loss from the under-to-aboveground transition due to the perpendicular propagation of the signal from higher to lower medium density (Dong & Vuran, 2013). Then, according to Dong & Vuran (2013), the path loss ℓ*ua*_*R*_*v*_*B*_, of the channel between *R*_*v*_, *v* ∈ [1, *N*_*R*_] and *B*, is obtained by adding the path losses ℓ*uu*_*R*_*v*_*G*_ and ℓ*aa*_*GB*_ for both underground and aboveground portions respectively, as follows

(3) \begin{eqnarray*}\ell {ua}_{{R}_{v}B}=\ell {uu}_{{R}_{v}G}+\ell {aa}_{GB}\end{eqnarray*}

with ℓ*uu*_*R*_*v*_*G*_ is given by

(4) \begin{eqnarray*}\ell {uu}_{{R}_{v}G}=6.4+20lo{g}_{10}({d}_{{R}_{v}G})+20lo{g}_{10}(\beta )+8.69\alpha {d}_{{R}_{v}G}\end{eqnarray*}

and ℓ*aa*_*GB*_ is given by

(5) \begin{eqnarray*}\ell {aa}_{GB}=-147.6+10\mu lo{g}_{10}({d}_{GB})+20lo{g}_{10}(q)\end{eqnarray*}

where *d*_*R*_*v*_*G*_ is equal to the burial depth of the relay node *R*_*v*_, ${d}_{GB}=\sqrt{{d}_{O{R}_{v}}^{2}+{d}_{OB}^{2}}$, with *d*_*OR*_*v*__ represents the distance between the origin *O* and *R*_*v*_, *d*_*OB*_ is the height of the aboveground base station *B*, and *μ* is the attenuation coefficient *via* air.

### Multi-relay UWSN communication

Here, the uplink communication procedure among nodes is presented. A time-division based transmission, and so, absence of co-channel interference are assumed. Each node *X* ∈ {*S*∪*N*_*R*_} is, then, assigned to one time slot in each packet transmission. Also, each node is characterized by a limited battery capacity *C*_*X*_ and, at each packet transmission, *t* ∈ [1, *τ*], it consumes a power ${P}_{X}^{t}$. To transmit a data packet *d*^*t*^, the process requires two phases. In the first phase, the source node *S* transmits the packet which reaches the set of relay nodes *R*_*v*_ ∈ [1, *N*_*R*_] located within the source communication range. The resulting received signal ${y}_{{R}_{v}}^{t}$ at each *R*_*v*_ is given by:

(6) \begin{eqnarray*}{y}_{{R}_{v}}^{t}=\sqrt{{P}_{S}^{t}}{h}_{S{R}_{v}}^{t}{d}^{t}+{n}_{{R}_{v}}^{t}\end{eqnarray*}

such that ${h}_{S{R}_{v}}^{t}$ represents the gain of the UG2UG channel between *S* and *R*_*v*_, which obeys the Rayleigh distribution (Vuran & Akyildiz, 2010) with the underground path loss ℓ*uu*_*SR*_*v*__ calculated in Eq. (1), and ${n}_{{R}_{v}}^{t}$ defines the zero-mean complex Additive White Gaussian Noise (AWGN) vector with variance *N*_0_. Then, in the second phase, ${N}_{R}^{S}$ selected cooperative relay nodes amplify and forward, each in a time slot, the signal ${y}_{{R}_{v}}^{t}$ to *B*. The proposed relay selection scheme will be presented in the next section. The received signal ${y}_{B}^{t}$ at *B* is calculated as follows:

(7) \begin{eqnarray*}{y}_{B}^{t}={A}_{v}^{t}\sqrt{{P}_{{R}_{v}}^{t}}{h}_{{R}_{v}B}^{t}{y}_{{R}_{v}}^{t}+{n}_{B}^{t}\end{eqnarray*}

where ${h}_{{R}_{v}B}^{t}$ is the UG2AG distributed Rayleigh channel between *R*_*v*_ and *B* (Vuran & Akyildiz, 2010) with the underground path loss ℓ*ua*_*R*_*v*_*B*_ calculated in Eq. (3), ${n}_{B}^{t}$ is the zero-mean complex AWGN vector, and ${A}_{v}^{t}= \frac{1}{\sqrt{{P}_{S}^{t}{|}{h}_{S{R}_{v}}^{t}{{|}}^{2}+{N}_{0}}} $ is the amplification factor. Since the received signals from the ${N}_{R}^{S}$ time slots are affected by different channel transfer function, the base station *B* combines them using the Maximum Ratio Combining (MRC) to compute the decision variable. As a result, the SNR at the output of the MRC, received at *B*, is expressed as the sum of the individual instantaneous SNRs of the two-hop links $(S,{R}_{v},B),{R}_{v}\in [1,{N}_{R}^{S}]$ and is given by

(8) \begin{eqnarray*}{{\Gamma }_{MRC}}^{t}=\sum _{v=1}^{{N}_{R}^{S}}{{\Gamma }_{S,{R}_{v},B}}^{t}=\sum _{v=1}^{{N}_{R}^{S}} \frac{{{A}_{v}^{t}}^{2}{P}_{{R}_{v}}^{t}{P}_{S}^{t}{|}{h}_{{R}_{v}B}^{t}{{|}}^{2}{|}{h}_{S{R}_{v}}^{t}{{|}}^{2}}{b{N}_{0}({{A}_{v}^{t}}^{2}{P}_{{R}_{v}}^{t}{|}{h}_{{R}_{v}B}^{t}{{|}}^{2}+1)} \end{eqnarray*}

where *b* denotes the channel bandwidth in Hz, which is, here, equal to the operating frequency *q*. Then, the maximum data rate of the cooperative network, denoted by *R*^*t*^, is given by

(9) \begin{eqnarray*}{R}^{t}=b{\log \nolimits }_{2}(1+{{\Gamma }_{MRC}}^{t}).\end{eqnarray*}

Hence, the energy efficiency *EE*^*t*^, defined as the total bits generated per unit energy consumed by sensor and relay nodes, equals

(10) \begin{eqnarray*}E{E}^{t}= \frac{{R}^{t}}{{P}_{S}^{t}+\sum _{v=1}^{{N}_{R}^{S}}{P}_{{R}_{v}}^{t}} .\end{eqnarray*}

Moreover, the spectral efficiency *SE*^*t*^, which represents the total delivered bits per unit bandwidth, equals

(11) \begin{eqnarray*}S{E}^{t}= \frac{{R}^{t}}{b} .\end{eqnarray*}

## Optimization Problem

In this article, we aim to jointly optimize the source and relay nodes power problem and to select the efficient set of cooperative relay node for a packet transmission. Indeed, the source and relay nodes powers ${P}_{X}^{t},X\in \{ S\cup {N}_{R}\} $ which maximize the resource efficiency metric *RE*^*t*^ are optimized. This metric guarantees an efficient balance between two competing metrics: energy efficiency *EE*^*t*^ and spectral efficiency *SE*^*t*^ at each packet transmission *t* ∈ [1, *τ*] as discussed in Tang et al. (2014). The resource efficiency metric *RE*^*t*^is given by

(12) \begin{eqnarray*}R{E}^{t}=E{E}^{t}+\omega S{E}^{t}\end{eqnarray*}

where $\omega =\bar {\omega } \frac{b}{{P}^{tot}} $ is the weighted factor with $\bar {\omega }$ is a constant and ${P}^{tot}={{P}_{S}}_{max}+{\mathop{\sum }\nolimits }_{v=1}^{{N}_{R}^{S}}{{P}_{{R}_{v}}}_{max}$ is the total power budget allocated to nodes. The considered optimization problem is formulated as finding the optimal source and relay node powers ${P}_{X}^{{t}^{opt}},X\in \{ S\cup {N}_{R}\} $ at each transmission *t* ∈ [1, *τ*] which depend on their limited battery capacity *C*_*X*_, their spent powers in previous transmissions $[{P}_{X}^{1},\ldots ,{P}_{X}^{t-1}]$ and the allowed power limitation range for each transmission [*P*_*X*_*min*__, *P*_*X*_*max*__]. Then, the proposed optimization problem is stated as finding ${P}_{X}^{{t}^{opt}}$ such that:

\begin{eqnarray*}\text{Max}~RE=\sum _{t=1}^{\tau }R{E}^{t} \end{eqnarray*}

\begin{eqnarray*}\text{Subject to}(\sum _{t=1}^{\tau }{P}_{X}^{t})\leq {C}_{X}, \end{eqnarray*}

(13) \begin{eqnarray*}\text{where}{P}_{X}^{t}\in [{P}_{{X}_{min}},{P}_{{X}_{max}}]\text{for}~X\in \{ S\cup {N}_{R}\} .\end{eqnarray*}

To improve the load balancing among relay nodes and the energy consumption of the whole network, a selected set of cooperative relay nodes ${N}_{R}^{S}$, from all eventual relay nodes *N*_*R*_ located within the source *S* communication range, is searched. The considered problem is a NP-hard multi-dimensional problem which can be solved efficiently using an optimization algorithm based on meta-heuristic approach detailed in the following section.

## Proposed Hybrid Chaotic Salp Swarm with Crossover Algorithm with Relay selection (HCSSC-RC)

In this section, we detail the proposed scheme for the considered multi-relay UWSN. First, the optimal source and relay nodes powers maximizing the resource efficiency *RE*^*t*^ at each packet transmission *t* are computed. Indeed, we propose to adapt the meta-heuristic HCSSC algorithm, proposed by Ayedi, ElAshmawi & Eldesouky (2022) for one relay system, to the multi-relay system. Then, we explain the relay nodes selection scheme. Assuming that *B* has perfect Channel State Information (CSI) awareness, the proposed algorithm is implemented at *B*, which will send the obtained optimal power values to the source and selected relay nodes prior to their packets transmissions.

### Hybrid chaotic salp swarm with crossover algorithm

The meta-heuristic HCSSC algorithm, proposed in Ayedi, ElAshmawi & Eldesouky (2022), is based on the the Salp Swarm Algorithm (SSA) which is one of the recent swarm algorithms proposed in Mirjalili et al. (2017) and is widely used in solving many optimization problems (Abualigah et al., 2020). The SSA emulates the motion of Salpidae that possess a limpid barrel-shaped body and live in deep oceans (Madin, 1990). Salps are organized in the form of a swarm called a salp chain. Each salp *x*^*i*^, *i* ∈ [1, *N*] in the algorithm population corresponds to a possible solution for the optimization problem where *N* is the total number of salps and *M* is the dimension of the salp which equals to the number of variables to optimize. The salp chain is divided mathematically into two groups: a leader, *x*^1^, which is the first salp of the chain, and followers, *x*^*i*^, *i* ∈ [2, *N*], which are the remaining salps that follow the leader. Since the global optimal solution of any optimization problem is unknown, the best solution can be obtained by moving the swarm leader, followed by the followers, towards the food source. As a result, the whole salp chain moves towards the global optimum.

For our considered problem, the resource efficiency optimization in the considered multi-relay UWSN is modelled as a multi-dimensional optimization problem with the maximized objective function is given in Eq. (12). Each salp ${x}^{i}=({x}_{1}^{i},\ldots ,{x}_{M}^{i})$ has *M* = *N*_*R*_ + 1 variables ${x}_{j}^{i},j\in [1,M]$ which are the transmission powers of source and relay nodes used at each transmission *t*. Thus, ${x}^{i}=({x}_{1}^{i},\ldots ,{x}_{M}^{i})=({P}_{S}^{t},{P}_{{R}_{1}}^{t},\ldots ,{P}_{{R}_{{N}_{R}}}^{t})$. The value of each variable ${x}_{j}^{i}={P}_{X}^{t}$ is searched within the lower and upper bounds *lb*_*j*_ = *P*_*X*_*min*__ and *ub*_*j*_ = *P*_*X*_*max*__.

#### Chaotic logistic map integration

The diversity of the initial population has a great impact on spreading effectively the salps initial positions in the search space. Therefore, to generate an effective initial population, the chaotic logistic map is used in the proposed algorithm as it is one of the simplest maps that exists in the nonlinear dynamics of a biological population and demonstrates the chaotic behaviour which guarantees the diversity of the individuals and enhances the search capability (Tang, Fong & Dey, 2018, Bingol & Alatas, 2020). Mathematically, the chaotic number is given by

(14) \begin{eqnarray*}{c}_{k+1}=r{c}_{k}(1-{c}_{k})\end{eqnarray*}

where *c*_*k*_ is the chaotic value at each independent run *k* (*i.e., c*_*k*_ ∈ (0, 1), and *r* is the growth rate that controls the behaviour of the chaotic value at a certain time (*r* =4). Accordingly, the initial population ${x}_{j}^{i},i\in [1,N],j\in [1,M]$ of the HCSSC algorithm is generated as follows

(15) \begin{eqnarray*}{x}_{j}^{i}=l{b}_{j}+{c}_{k}(u{b}_{j}-l{b}_{j}).\end{eqnarray*}

In SSA, the update computation of the position of the leader is based on random coefficients *c*_2_ and *c*_3_ which direct the movement of the leader towards a positive or negative infinity. Hence, the chaotic logistic map is also employed to compute these numbers for better exploration of the search space. The integration of the chaotic map in the SSA algorithm is well detailed in Ayedi, ElAshmawi & Eldesouky (2022).

#### Crossover technique

Each follower *x*^*i*^, *i* ∈ [2, *N*] in the population updates its position based on how far the current position is from the best salp’s position. Since the uniform crossover proves its efficiency compared to other crossover operators (Syswerda et al., 1989; Hussain, Muhammad & Sajid, 2018; Umbarkar & Sheth, 2015), authors in Ayedi, ElAshmawi & Eldesouky (2022) propose to integrate it in the salps positions updates for one-relay network. In the following, we detail the application of the crossover technique in case of multi-relay UWSN.

Each current individual $a=({a}_{1}^{i},\ldots ,{a}_{M}^{i})$ along with the actual best individual $b=({b}_{1}^{i},\ldots ,{b}_{M}^{i})$, where *M* = *N*_*R*_ + 1, are nominated as parents. The individuala *a* and *b* are mate using a binary mask, having the same length *M* as well. The mask consists of binary digits and is generated randomly. The digits of two offsprings, $\widetilde {a}$ and $\widetilde {b}$, are duplicated from parents as per the bits of the mask. For the first offspring $\widetilde {a}$, if the digit in a mask is 1, then the digit is taken from *a*, otherwise, it is taken from *b*. For the second offspring $\widetilde {b}$, the complement of the mask is used. The two obtained offsprings $\widetilde {a}$ and $\widetilde {b}$, are considered as two new salps in the population. Then, the objective function, which is the resource efficiency, *RE*^*t*^ given in Eq. (12), of each offspring, is evaluated. The offspring having the highest resource efficiency is chosen to be *x*_*ucross*_. Consequently, each follower in the salp chain updates its position as follows

(16) \begin{eqnarray*}{x}_{j}^{i}= \frac{1}{2} ({x}_{ucross}+{x}_{j}^{i-1}) \forall i\geq 2\end{eqnarray*}

**Figure 2:**
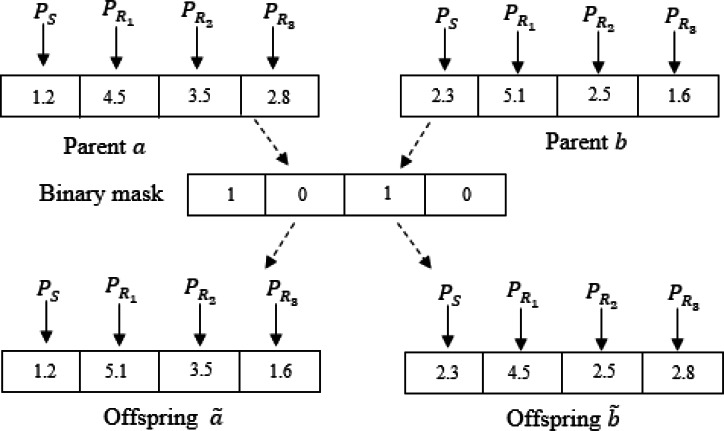
An llustrative example of the uniform crossover for *N*_*R*_ = 3.

Figure 2 illustrates an illustrative example of the uniform crossover for *N*_*R*_ = 3. The leader and followers update their positions iteratively until reaching the maximum number of iterations *IT*_*max*_. Hence, the optimal values of the source and relay nodes powers are obtained.

### Relay selection approach

Having obtained the optimal powers for *N*_*R*_ relay nodes, the base station *B* decides which relay nodes should actually participate in the data transmission by comparing ${P}_{{R}_{v}}^{{t}^{opt}},{R}_{v}\in {N}_{R}$ to a given threshold *γ* and feeds back the CSI to those nodes. If ${P}_{{R}_{v}}^{{t}^{opt}}\geq \gamma $, the relay is selected to cooperate. Otherwise, the relay node is not selected and his power is set to 0. This criteria ensures that only the most beneficial relay nodes to the communication performance are selected and simultaneously, the total consumed power is minimized as the number of cooperating relays is limited. Generally, the determination of the value of this threshold is based on the designer objective related to the available resources in terms of bandwidth and power. The impact of this threshold adjustment is studied in Section 5.

**Figure 3:**
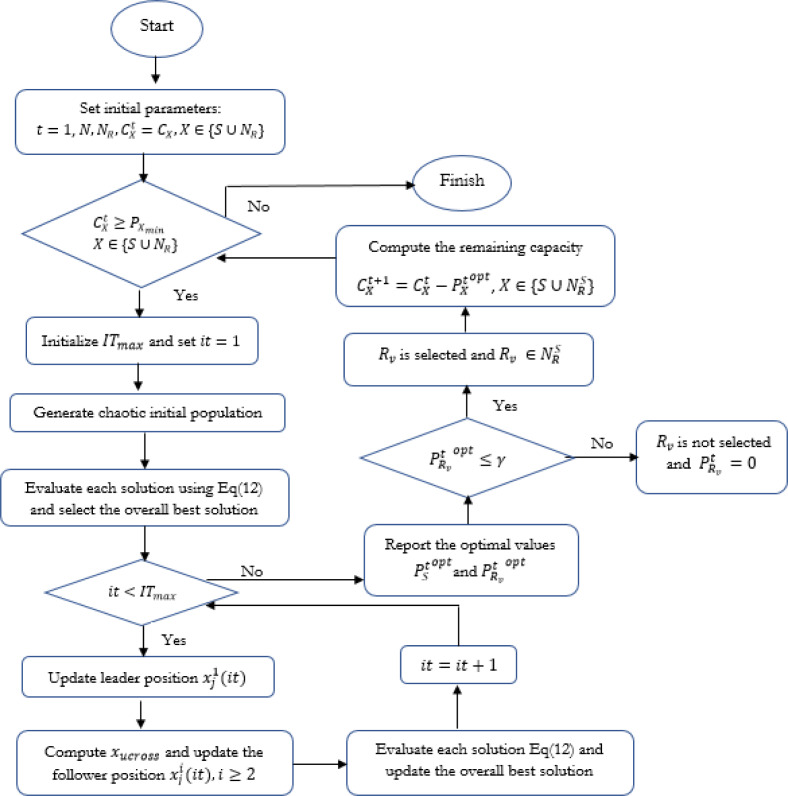
Flowchart of the proposed HCSSC-RC algorithm.

The overall algorithm for optimizing the resource efficiency based on the HCSSC-RC algorithm is described in Fig. 3.

## Experimental Results and Analysis

This section presents the experimental results illustrating the performance of the proposed power optimization algorithm. Simulation results are obtained by averaging over 1,000 channel iterations. We assume that nodes *X* ∈ {*S*∪*N*_*R*_} have equal battery capacities *C*_*X*_ = *C* = 3*w*, equal minimum allowed powers *P*_*X*_*min*__ = *P*_*min*_ = 5 mw and equal maximum allowed powers *P*_*X*_*max*__ = *P*_*max*_. The authors in Ayedi, Eldesouky & Nazeer (2021) detailed the UG2UG and UG2AG path losses parameters specifications. The number of relay nodes located within the source communication range is *N*_*R*_ = 5. Coordinates, in meters (m), of the source node, are *S*^*x*^ = 0.2 m, *S*^*y*^ = 0.2 m and *S*^*z*^ = 0.5 m. The height *h*_*B*_ of the base station *B* is set to 0.7 m.

The improvement of the resource efficiency obtained using the proposed HCSSC-RS algorithm, *RE*_*HCSSC*−*RS*_, against the resource efficiency, obtained when using any other meta-heuristic algorithm *A* applied with with relay nodes selection *RE*_*A*−*RS*_, is computed as follows

(17) \begin{eqnarray*}\text{improvement}~(\text{%})= \frac{R{E}_{HCSSC-RS}-R{E}_{A-RS}}{R{E}_{A-RS}} \times 100.\end{eqnarray*}

Table 1 shows the obtained average number of selected cooperative relay nodes ${N}_{R}^{S}$ for various values of the threshold *γ* and various maximum allowed power *P*_*max*_. Obviously, the number of cooperative relay nodes minimizes when *γ* is approaching *P*_*max*_.

**Table 1:** Average number of selected cooperative relay nodes for different values of *γ* and *P*_*max*_.

*P*_*max*_(*mw*)	*γ*(*mw*)	Average ${N}_{R}^{S}$
50	49	2
	35	3
	6	4
	5	5
100	98	2
	50	3
	7	4
	5	5
150	147	2
	75	3
	7	4
	5	5
200	196	2
	100	3
	8	4
	5	5
250	243	2
	125	3
	9	4
	5	5

**Figure 4:**
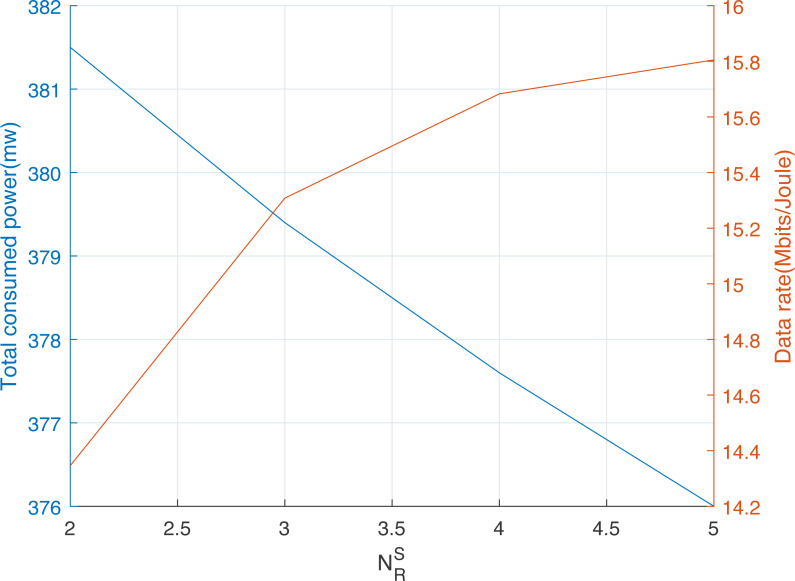
Data rate and total consumed power using the HCSSC-RS *versus* the number of selected cooperative relay nodes ${N}_{R}^{S}$.

Figure 4 shows the effect of threshold *γ*, and hence, the number of selected relay nodes ${N}_{R}^{S}$ on the total consumed power and the obtained data rate for *P*_*max*_ = 200 mw. Evidently, the augmentation of the number of cooperative relay nodes enhances the diversity gain and hence the network data rate but increases the total consumed power. In addition, the value of *γ* should be adjusted depending on the desired level of the network data rate given the total consumed power constraint.

**Figure 5:**
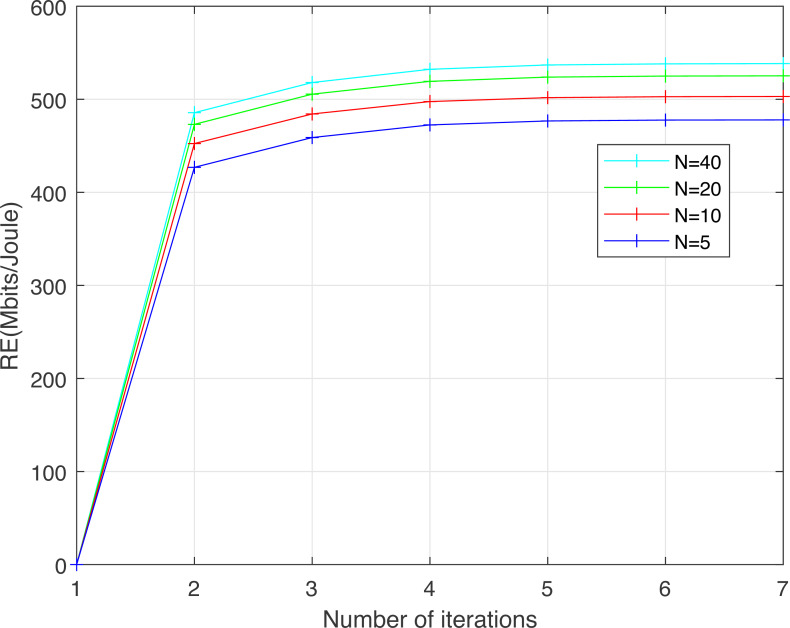
Convergence curve of the resource efficiency obtained using the proposed algorithm *versus* the number of iterations for different numbers of salps *N*.

Figure 5 shows the convergence, as a function of the number of iterations, of the resource efficiency *RE* obtained using the proposed HCSSC-RS for various numbers of salps *N* ∈ {5, 10, 20, 40}. The number of cooperative relay nodes ${N}_{R}^{S}$ is set to 3. The maximum nodes transmission power *P*_*max*_ is set to 200 mw. Clearly, the convergence of the proposed algorithm is rapidly obtained for various numbers of salps to reach the optimal *RE* values. Additionally, the increase in the number of salps enhances the computational accuracy of the optimal *RE*. Hence, the proposed algorithm can be efficiently implemented with an acceptable cost of computational complexity. We set the salps number *N* to 20 in all following simulation results.

**Figure 6:**
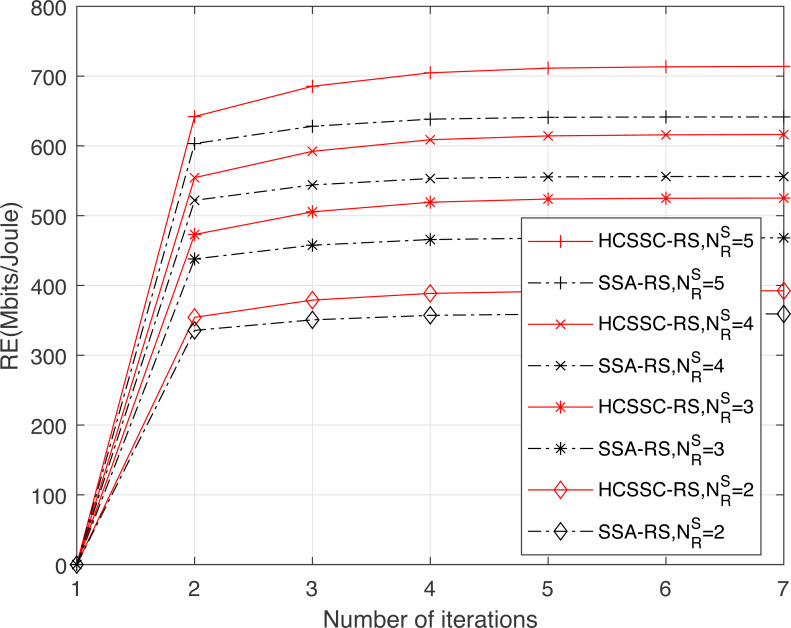
Convergence curves of the resource efficiency obtained using the proposed algorithm HCSSC-RS and the algorithm SSA-RS *versus* the number of iterations for different values of threshold *γ*.

Figure 6 illustrates the convergence of the resource efficiency obtained using the HCSSC-RS and SSA-RS for different threshold values which gives different number of selected relay nodes ${N}_{R}^{S}\in \{ 2,3,4,5\} $ for *P*_*max*_ = 200 mw as shown in Table 1. Clearly, the proposed algorithm achieves higher resource efficiency compared to the standard SSA. Indeed, the generation of initial population using chaotic map enhances the exploration of the search space and, consequently, gives better results in maximizing the resource efficiency, compared to the random population initialization used in SSA, during the number of iterations. Moreover, the crossover operation ameliorates the search of the optimal solution and may prevent the premature convergence especially by the generation of the offsprings. As shown from the figure, the gain gap between the HCSSC and SSA based schemes increases as the number of selected relay nodes increases which confirms the efficiency of the proposed algorithm for high dimensional problems. In fact, the improvement reaches 9.2% for ${N}_{R}^{S}=2$ and reaches 11.3% for ${N}_{R}^{S}=5$.

**Figure 7:**
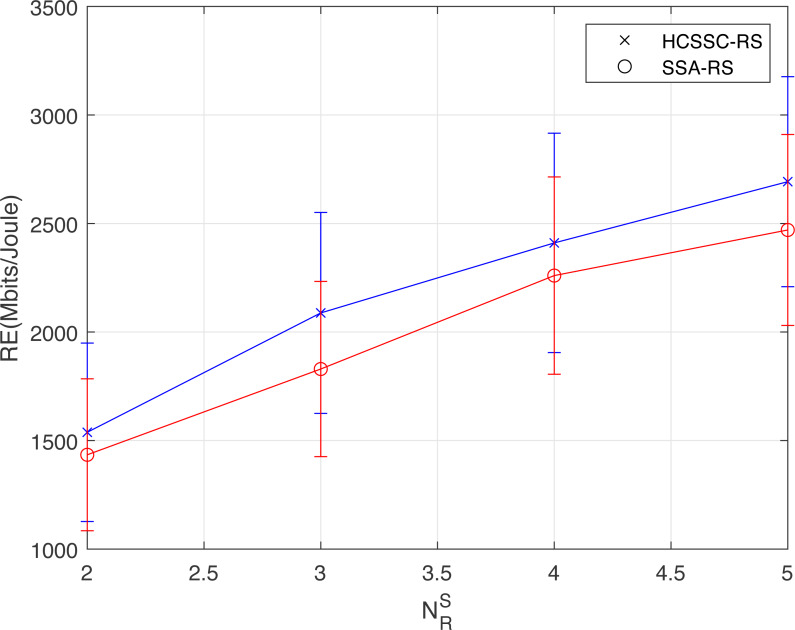
Statistical results of *RE*(*Mbits*/*Joule*) obtained using the HCSSC-RS and SSA-RS for different number of selected cooperative relay nodes ${N}_{R}^{S}$.

Table 2 and Fig. 7 illustrate the maximum (Max.), minimum (Min.), average (Avg.), and standard deviation (Std.) of *RE* for various number of selected relay nodes numbers ${N}_{R}^{S}$. The maximum allowed power *P*_*max*_ = 50 mw. The proposed HCSSC-RC based optimization scheme outperforms the SSA-RC based scheme in all evaluated performance measurements. For example, when ${N}_{R}^{S}=3$, the maximum value of the HCSSC-RS based scheme reaches 2550.92 Mbits/Joule, while the maximum value of the SSA-RC based scheme reaches 2233.36 Mbits/Joule. Hence, the gain is approximately 14.21%.

**Figure 8:**
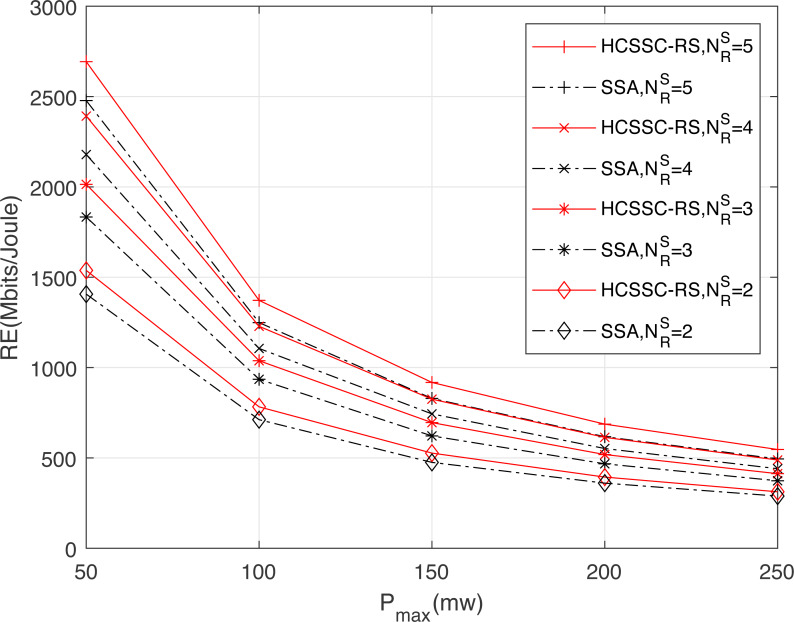
Resource efficiency obtained using the HCSSC-RS and SSA-RS *versus* the maximum allowed power *P*_*max*_ for different number of selected cooperative relay nodes ${N}_{R}^{S}$.

Figure 8 illustrates the effect of the maximum power permitted for a transmission on resource efficiency for both algorithms and for different relay nodes number. Compared to the SSA-RC, the HCSSC-RC achieves a higher resource efficiency at equal power cost. Indeed, the combination of chaotic map and the cross over operations in the proposed power algorithm improves the optimal nodes powers search considering the physical power limitations. Here again, the gain gap between the HCSSC and the SSA schemes is higher as ${N}_{R}^{S}$ increases. In fact, for ${N}_{R}^{S}=2$, the improvement reaches 9.33% at *P*_*max*_ = 50 mw and reaches 10.81% at *P*_*max*_ = 150 mw. Moreover, for ${N}_{R}^{S}=5$, the gain in *RE* is 10.4% at *P*_*max*_ = 50 mw and is 11.9% at *P*_*max*_ = 150 mw. Since the increase in the maximum allowed power degrades the energy efficiency and the weighted spectral efficiency as well, it degrades, then, the resource efficiency. In addition, network designers should regulate the maximum power depending on the available number of cooperative relays for a given resource efficiency specification.

**Figure 9:**
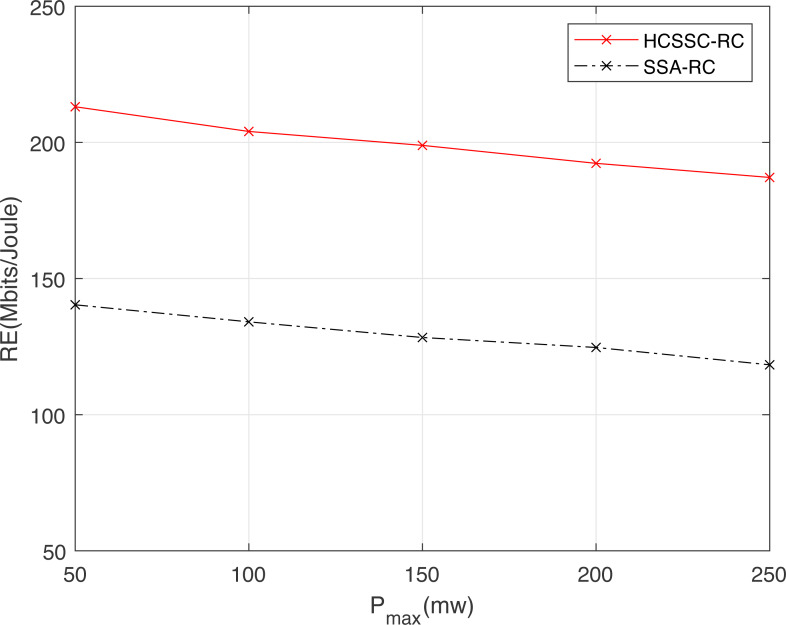
Resource efficiency obtained using the HCSSC-RS and SSA-RS *versus* the maximum allowed power *P*_*max*_ in case of variable number of selected cooperative relay nodes among transmissions.

In Fig. 9, we propose to study the case where the value of the threshold *γ*, i.e, the number of selected relay nodes changes among different transmissions. In fact, some relay nodes can not cooperate in some periods due to the battery drain or to the communication interruption caused by the harsh environmental conditions. For this purpose, we consider that only six successive transmissions, *t* ∈ [1, 6], are performed and the value of *γ* variates from one transmission to another resulting to the variation of the number of cooperative relays as shown in Table 3. Figure 9, illustrates the resource efficiency obtained using HCSSC-RS compared to that obtained using SSA-RS *versus* the maximum allowed power *P*_*max*_. We, clearly, remark the improvement in resource efficiency when HCSSC-RS is used compared to the SSA-RS for all allowed maximum power.

**Table 2:** Statistical results of *RE*(*Mbits*/*Joule*) for various relay nodes numbers with *P*_*max*_ = 50 *mw*.

	SSA-RS				HCSSC-RS			
${N}_{R}^{S}$	Avg.	Max.	Min.	Std.	Avg.	Max.	Min.	Std.
2	1410.12	1784.55	1084.48	114.80	1532.23	1949.14	1127.21	128.06
3	1834.06	2233.36	1425.91	120.96	2018.33	2550.92	1625.11	136.19
4	2185.85	2714.47	1805.66	129.91	2391.34	2915.85	1905.24	144.67
5	2465.46	2909.94	2030.34	141.72	2690.22	3176.44	2208.87	143.96

**Figure 10:**
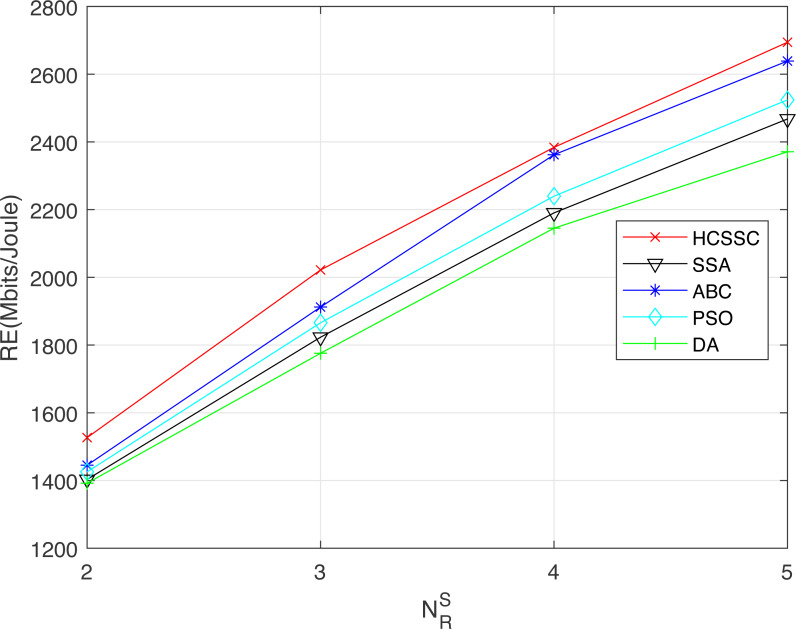
Resource efficiency obtained using the algorithms: HCSSC, SSA, PSO, ABC and DA *versus* the number of selected cooperative relay nodes ${N}_{R}^{S}$.

**Figure 11:**
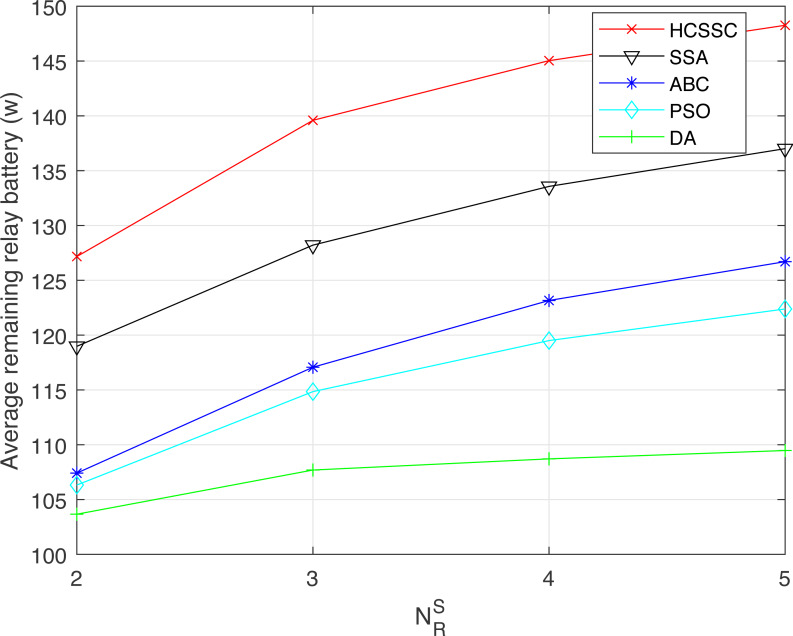
Average remaining relay battery obtained using the algorithms: HCSSC, SSA, PSO, ABC and DA *versus* the number of selected cooperative relay nodes ${N}_{R}^{S}$.

**Figure 12:**
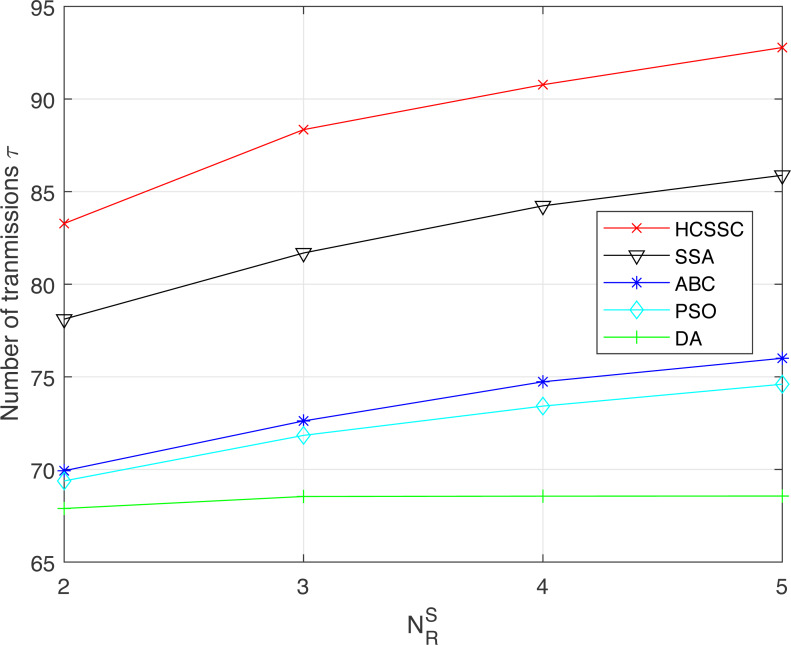
Number of transmissions performed using the algorithms: HCSSC, SSA, PSO, ABC and DA *versus* the number of selected cooperative relay nodes ${N}_{R}^{S}$.

To demonstrate the efficiency of using the chaos theory and the crossover operator in case of multi-relay system, the HCSSC algorithm is compared to other meta-heuristic algorithms, which are SSA, PSO, ABC and DA, in terms of resource efficiency, average remaining relay battery and performed number of transmissions in Figs. 10 and 11 and 12 respectively. The maximum power *P*_*max*_ is set to 50 mw.

Figure 10 illustrates the network resource efficiency for all algorithms. It shows that HCSSC offers the highest resource efficiency among all other meta-heuristics algorithms. For example, when ${N}_{R}^{S}=5$, the proposed algorithms achieves a gain of 3.5% compared to ABC, 7.4% compared to PSO, 12.4% compared to SSA and 20.8% compared to DA. Furthermore, the results confirm the exceeding of the proposed algorithm with the increase of the number of relay nodes which means the increase of number of the problem variables.

Figure 11 shows the average remaining relay battery per relay accumulated over all performed transmissions for all algorithms. We notice that the proposed HCSSC significantly enhances the average remaining relay battery value, for all relay nodes number, compared to all other algorithms. Moreover, the gap is higher when ${N}_{R}^{S}$ increases. The improvement performance in the average remaining relay battery can be computed by replacing *RE* in Eq. (17). When ${N}_{R}^{S}=2$, the obtained gain is 7.6% compared to SSA, 18.3% compared to ABC, 19.6% compared to PSO and 22.6% compared to DA. When ${N}_{R}^{S}=5$, the gain reaches 8% compared to SSA, 19.2% compared to ABC, 21.2% compared to PSO and 37.1% compared to DA. Additionally, the proposed algorithm based power optimization scheme extends the nodes batteries lifetime.

Figure 12 presents the impact of the proposed HCSSC based power allocation scheme on the number of transmissions *τ* successfully performed compared to the other algorithms. Remarkably, the proposed algorithm provides the highest number of transmissions compared to all other algorithms. We also measure the improvement performance in the number of transmission by replacing *RE* in Eq. (17). When ${N}_{R}^{S}=2$, the improvement is 6.6% compared to SSA, 19% compared to ABC, 20% compared to PSO and 22.6% compared to DA. When ${N}_{R}^{S}=5$, the improvement reaches 8% compared to SSA, 22.2% compared to ABC, 24.7% compared to PSO and 37.6% compared to DA. Hence, the proposed algorithm based power optimization scheme enhances the network capacity and expands the network utilization specially when the number of cooperative relay nodes increases.

## Conclusion

In this article, we proposed an improved SSA algorithm for multi-relay UWSNs where multiple underground cooperative relay nodes help the sensor node to transmit collected data to the base station. The aim is to maximize the network resource efficiency which is a unified metric that jointly combines both energy and spectral efficiencies. The algorithm improves the standard meta-heuristic SSA by the use of logistic chaotic map in the generation of the initial population and the deployment of the uniform crossover operator in the salps positions updates. At each packet transmission, the proposed algorithm is applied to provide the optimal source and relay nodes powers maximizing the resource efficiency considering the remaining nodes batteries capacities with the lower and upper allowed powers constraints. Simulations showed that the integration of the chaotic map and the use of the crossover method enhanced the optimal solution computation and, then, improved the resource efficiency of the network compared to that obtained using the standard SSA. Moreover, the enhancement is significant when the number of cooperative relay nodes increases which means that the number of the problem variables increases. Also, the proposed algorithm proves its superiority to other meta-heuristic algorithms in maximizing the resource efficiency and extending the nodes batteries lifetime and the network longevity.

**Table 3:** Variation of selected relay nodes number among transmissions.

*t*	1	2	3	4	5	6
${N}_{R}^{S}$	4	4	3	3	2	2

## Supplemental Information

10.7717/peerj-cs.1357/supp-1 Supplemental Information 1 Resource efficiency optimization using the proposed algorithm For an UWSN consisting of a source node and five relay nodes, and given initial batteries with 3w, the proposed algorithm is applied to compute the optimal resource efficiency function at each packet transmission. The treatment is averaged over 1,000 iterations and repeated until batteries drains.

## Additional Information and Declarations

### Data Availability

The following information was supplied regarding data availability: The code is available in the Supplemental File.
